# Posture Does Not Matter! Paw Usage and Grasping Paw Preference in a Small-Bodied Rooting Quadrupedal Mammal

**DOI:** 10.1371/journal.pone.0038228

**Published:** 2012-05-30

**Authors:** Marine Joly, Marina Scheumann, Elke Zimmermann

**Affiliations:** 1 Institute of Zoology, University of Veterinary Medicine Hannover, Hannover, Germany; 2 Center for Systems Neuroscience, Hannover, Germany; California State University Fullerton, United States of America

## Abstract

**Background:**

Recent results in birds, marsupials, rodents and nonhuman primates suggest that phylogeny and ecological factors such as body size, diet and postural habit of a species influence limb usage and the direction and strength of limb laterality. To examine to which extent these findings can be generalised to small-bodied rooting quadrupedal mammals, we studied trees shrews *(Tupaia belangeri)*.

**Methodology/Principal Findings:**

We established a behavioural test battery for examining paw usage comparable to small-bodied primates and tested 36 *Tupaia belangeri*. We studied paw usage in a natural foraging situation (simple food grasping task) and measured the influence of varying postural demands (triped, biped, cling, sit) on paw preferences by applying a forced-food grasping task similar to other small-bodied primates. Our findings suggest that rooting tree shrews prefer mouth over paw usage to catch food in a natural foraging situation. Moreover, we demonstrated that despite differences in postural demand, tree shrews show a strong and consistent individual paw preference for grasping across different tasks, but no paw preference at a population level.

**Conclusions/Significance:**

Tree shrews showed less paw usage than small-bodied quadrupedal and arboreal primates, but the same paw preference. Our results confirm that individual paw preferences remain constant irrespective of postural demand in some small-bodied quadrupedal non primate and primate mammals which do not require fine motoric control for manipulating food items. Our findings suggest that the lack of paw/hand preference for grasping food at a population level is a universal pattern among those species and that the influence of postural demand on manual lateralisation in quadrupeds may have evolved in large-bodied species specialised in fine manipulations of food items.

## Introduction

First thought to be a unique trait in humans, a side bias in limb preference has been reported in a variety of tetrapod species (e.g. [1], [2], [3], [4], [5], [6]). While most of the results demonstrated that tested individuals have a side bias in their limb usage, supposed to reflect a cerebral lateralisation, a bias at the population level is not universal (e.g. [1], [6], [7], [8], [9], [10], [11]). Among tetrapod species, it has been shown that a side bias in limb usage at a population level seems to be constrained by phylogeny but also varies according to ecological variables such as body size, foraging mode and postural habit [12], [13], [14].

For instance, a recent analysis in 23 different Australian parrot species showed that while direction and strength of lateralisation are constrained by phylogeny, the strength of laterality varies between the species, probably due to a shift in foraging mode [12]. Larger bodied parrot species eat large seeds they have to extract from seedpods, requiring manipulation with a limb, and demonstrate strong lateralisation, while small-bodied parrot species which eat small seeds and blossom, requiring no manipulation with a limb, are nonlateralised [12]. Therefore, the relative costs and benefits associated with laterality are expected to vary depending on the physical environment in which animals live.

In mammals, numerous studies on motor asymmetries have been conducted in nonhuman primates (e.g. [11], [14], [15], [16], [17], [18], [19], [20], [21], [22], [23], [24], [25], [26], [27] but only few in non primate species (e.g. [1], [2], [5], [6], [13]). In nonhuman primates, it has been demonstrated that the direction and the strength of the side bias in an individual may be largely influenced by body posture (e.g. [14], [15], [16], [20], [23], [26], [27], [28], [29], [30], [31]). A tendency towards increasing the strength of hand preference from a stable reaching position (quadrupedal or sit) to an unstable reaching position (bipedal, cling) was found in some prosimians [18], [32], [33], some Old World monkeys [17], some New World monkeys [26], [34], [35] and some great apes [20], [36]. However, these results seem to vary between species independent of the phylogeny. Indeed, for instance within prosimians, Senegal bushbabies [32], [33] showed an increase in their strength of hand preference in bimanual versus quadrupedal tasks, whereas in gray mouse lemurs there was no effect of the postural demand on the strength of hand preference [14], [31]. The results on prosimians therefore suggest that the more vertical body orientation a species has (e.g. Senegal bushbabies are leapers and move from branch to branch more vertically than mouse lemurs which are quadruped and move horizontally), the greater the influence of the postural demand on the strength of manual laterality.

To the best of our knowledge, within non primate mammals, only 2 recent studies, one on cats [2] and the other on wallabies [13] assessed the influence of body posture on the paw preference. In cats, differences in postural demands (sit or cling) altered neither the direction nor the strength of paw preference [2] while wallabies showed a left preference and were more lateralised when feeding in a bipedal position than in a quadrupedal position [13]. The results of both studies in non primate mammals are therefore in line with the suggestion in nonhuman primates: the more biped a species is (i.e. wallabies *vs* cats in non primate mammals; bushbabies *vs* mouse lemurs in nonhuman primates), the stronger the bias to use one hand, and the greater the influence of the postural demand on the strength of manual laterality.

To examine to what extent findings on manual laterality and influence of body posture on manual laterality can be generalised within mammals, we tested tree shrews, as a model for small-bodied quadrupedal mammals. Tree shrews are omnivorous mammals and are classified into the order Scandentia. First considered as primates, they are now classified in their own order and placed together with primates and dermoptera within the clade Euarchonta [37]. As reviewed by Sargis, there is contradictory behavioural and morphological evidence regarding the grasping abilities of *Tupaia* [38], [39]. They have claws on all their fingers and their thumb is hardly different from the other digits. He states that grasping in *Tupaia* is not mechanically identical and “incipient” but similar when compared with that of primates. Due to similar grasping capabilities and their close phylogenetic relationships to primates, *Tupaia* therefore represents an interesting model for gaining insight into the ecological variables which may affect manual lateralisation in both non primates and nonhuman primates.

In our study, we aimed at characterising for the first time the paw usage of a small-bodied quadrupedal mammal *(Tupaia belangeri)*, both at an individual and population level, and at exploring the effect of different postural demands on the direction and strength of paw preference. Since Rogers suggested that results on handedness could vary depending on the assay applied [40], we tested for our comparative approach the tree shrews in a test battery comparable to other small-bodied quadruped primates [8], [14], [28].

As this tree shrew species mostly moves quadrupedally and shows rooting behaviour during foraging, we expect the animals to naturally catch food preferentially with the mouth than using paws in a simple food grasping task representing the natural foraging situation. Moreover, since tree shrews do also climb, we expect that they are able to use only one paw for grasping. We will herewith explore whether individuals and the tested population show paw preference while grasping for food. Lastly, when grasping in different postures (triped, biped, cling or sit), we expect the task in tripedal posture to be easier to solve for *T. belangeri* than the more unstable bipedal, cling or sit postures. We will investigate whether the postural demand influences the direction and strength of paw preference for grasping. The results will be discussed according to possible evolutionary scenarios of the paw/hand usage, paw/hand preference and influence of postural demand on paw/hand preference in mammals.

## Results

### Simple food grasping task (SGT)

In the SGT task, all tree shrews (N = 14) mostly used the mouth alone to grasp a mealworm. Only 1 subject used a paw-mouth combination 3 times to grasp a mealworm (2 times with the right and once with the left paw). No grasping with only one paw was observed.

### Postural tasks

In the FGT-triped task, 29 of the subjects (N = 36; 80.6%; Table 1) showed an individual paw preference by using one paw significantly more often than the other (binominal test: p≤0.05): 14 subjects were right-pawed and 15 subjects were left-pawed. The number of lateralised subjects was significantly higher than expected by chance (Chi-Square = 13.5, df = 2, N = 36, p<0.001). No population level paw preference was found since the number of left- and right-pawed subjects was not significantly different from chance (Binomial test: p = 1.0). A one-sample t-test indicated that the mean PI_triped_ score per subject (mean_triped_ = −0.09, SD = 0.84) did not differ significantly from chance (one-sample t-test: t = −0.611, df = 35, p = 0.545).

**Table 1:** Pawedness Index (PI) and pawedness bias for each subject and each postural task.

Subject	Sex	Age	FGT-TripedPI (bias)	FGT- BipedPI (bias)	FGT-ClingPI (bias)	FGT-SitPI (bias)
**Daisy**	**f**	**1**	**−1.00 (L)**	**−1.00 (L)**	**−1.00 (L)**	**−1.00 (L)**
**Eowyn**	**f**	**5**	**−1.00 (L)**	**−1.00 (L)**	**−1.00 (L)**	**−1.00 (L)**
**Lilli**	**f**	**5**	**−1.00 (L)**	**−1.00 (L)**	**−1.00 (L)**	**−1.00 (L)**
**Rosi**	**f**	**5**	**−1.00 (L)**	**−1.00 (L)**	**−1.00 (L)**	**−1.00 (L)**
Nele	f	4	−1.00 (L)	0.19 (A)	−0.05 (A)	−0.05 (A)
**Pia**	**f**	**1**	**−0.96 (L)**	**−0.95 (L)**	**−1.00 (L)**	**−1.00 (L)**
**Anna**	**f**	**5**	**−0.95 (L)**	**−0.74 (L)**	**−0.49 (L)**	**−0.82 (L)**
**Maja**	**f**	**6**	**−0.89 (L)**	**−1.00 (L)**	**−1.00 (L)**	**−1.00 (L)**
Selma	f	6	−0.06 (A)	0.06 (A)	0.03 (A)	0.56 (R)
Berta	f	1	0.13 (A)	0.73 (R)	0.33 (A)	0.05 (A)
Beatrice	f	1	0.56 (R)	−0.56 (L)	−0.94 (L)	−1.00 (L)
**Ilse**	**f**	**5**	**0.67 (R)**	**0.90 (R)**	**1.00 (R)**	**1.00 (R)**
Gretchen	f	6	0.86 (R)	0.54 (R)	−0.03 (A)	0.77 (R)
**Paula**	**f**	**1**	**0.90 (R)**	**1.00 (R)**	**1.00 (R)**	**1.00 (R)**
**Bea**	**f**	**4**	**1.00 (R)**	**1.00 (R)**	**1.00 (R)**	**1.00 (R)**
**Dolly**	**f**	**4**	**1.00 (R)**	**1.00 (R)**	**1.00 (R)**	**1.00 (R)**
**Idefix**	**m**	**4**	**−1.00 (L)**	**−1.00 (L)**	**−1.00 (L)**	**−1.00 (L)**
**Abel**	**m**	**6**	**−1.00 (L)**	**−0.96 (L)**	**−0.83 (L)**	**−0.95 (L)**
**Aragorn**	**m**	**6**	**−1.00 (L)**	**−0.96 (L)**	**−1.00 (L)**	**−1.00 (L)**
Piet	m	1	−0.89 (L)	−0.12 (A)	−0.07 (A)	−0.07 (A)
Barbossa	m	1	−0.25 (A)	−0.27 (A)	−0.17 (A)	−0.51 (L)
Pluto	m	1	−0.11 (A)	−0.73 (L)	−0.67 (L)	−0.76 (L)
Don	m	1	0.24 (A)	0.41 (R)	0.42 (R)	−0.77 (L)
Frodo	m	5	0.50 (R)	0.59 (R)	0.21 (A)	0.79 (R)
**Pelle**	**m**	**1**	**0.87 (R)**	**1.00 (R)**	**1.00 (R)**	**1.00 (R)**
Isidor	m	6	0.94 (R)	0.68 (R)	0.23 (A)	0.45 (R)
**Isegrim**	**m**	**5**	**1.00 (R)**	**1.00 (R)**	**0.90 (R)**	**1.00 (R)**
**Goofy**	**m**	**4**	**1.00 (R)**	**1.00 (R)**	**0.96 (R)**	**1.00 (R)**
Clara	f	3	−0.93 (L)	−1.00 (L)	−1.00 (L)	
Dora	f	5	−0.03 (A)	0.67 (R)	0.63 (R)	
Hugo	m	9	−1.00 (L)	−1.00 (L)	−1.00 (L)	
Omo	m	4	−1.00 (L)	−1.00 (L)	−0.96 (L)	
Nemo	m	5	−0.08 (A)	0.94 (R)	0.94 (R)	
Oskar	m	4	1.00 (R)	1.00 (R)	1.00 (R)	
Otto	m	4	1.00 (R)	1.00 (R)	1.00 (R)	
Omar	m	4	0.41 (R)	0.89 (R)		0.85 (R)

For each subject, the sex (m: male and f: female) and age (in years) are given. PI and bias are indicated for each postural task. L indicates a left bias, R a right bias and A means ambidextrous. Bold marked subjects showed consistent paw preference for all four postural tasks.

In the FGT-biped task, 32 of the subjects (N = 36; 88.9%; Table 1) showed an individual paw preference by using one paw significantly more often than the other (Binominal test: p≤0.05): 17 subjects were right-pawed and 15 subjects were left-pawed. The number of lateralised subjects was significantly higher than expected by chance (Chi-Square = 22, df = 2, N = 36, p<0.0001). No population level paw preference was found since the number of left- and right-pawed subjects was not different from chance (binomial test: p = 0.860). A one-sample t-test indicated that the mean PI_biped_ score per subject (mean_biped_ = 0.009, SD = 0.862) did not differ significantly from chance (one-sample t-test: t = 0.06, df = 35, p = 0.953).

In the FGT-cling task, 27 of the subjects (N = 35; 77.1%; Table 1) showed an individual paw preference by using one paw significantly more often than the other (Binominal test: p≤0.05): 12 subjects were right-pawed and 15 subjects were left-pawed. The number of lateralised subjects was significantly higher than expected by chance (Chi-Square = 10.829, df = 2, N = 35, p = 0.004). No population level paw preference was found since the number of left- and right-pawed subjects was not different from chance (Binomial test: p = 0.701). A one-sample t-test indicated that the mean PI_cling_ score per subject (mean_cling_ = −0.0731, SD = 0.830) did not differ significantly from chance (one-sample t-test: t = −0.521, df = 34, p = 0.606).

In the FGT-sit task, 26 of the subjects (N = 29; 89.7%; Table 1) showed an individual paw preference by using one paw significantly more often than the other (binominal test: p≤0.05): 12 subjects were right-pawed and 14 subjects were left-pawed. The number of lateralised subjects was significantly higher than expected by chance (Chi-Square = 18.5, df = 2, N = 29, p<0.001). No population level paw preference was found since the number of left- and right-pawed subjects was not different from chance (Binomial test: p = 0.845). A one-sample t-test indicated that the mean PI_sit_ score per subject (mean_sit_ = −0.085, SD = 0.871) did not differ significantly from chance (t = −0.524, df = 28, p = 0.604).

In each task, we found no significant difference in the PI and ABS-PI between the sexes (Mann-Whitney-U: p≥0.229) and also no correlation between age and PI or ABS-PI (Spearman correlation: p≥0.300).

### Comparison of postural tasks

**Figure 1:**
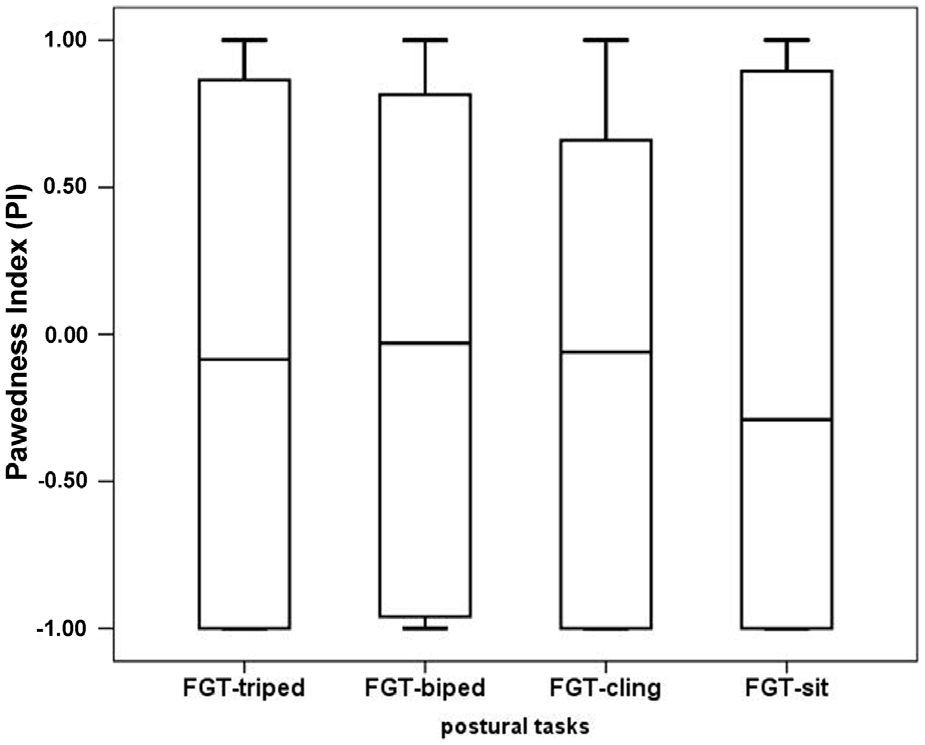
Mean Pawedness Index (PI) for the four postural tasks. The same individuals were tested in all four tasks (N = 28).

**Figure 2:**
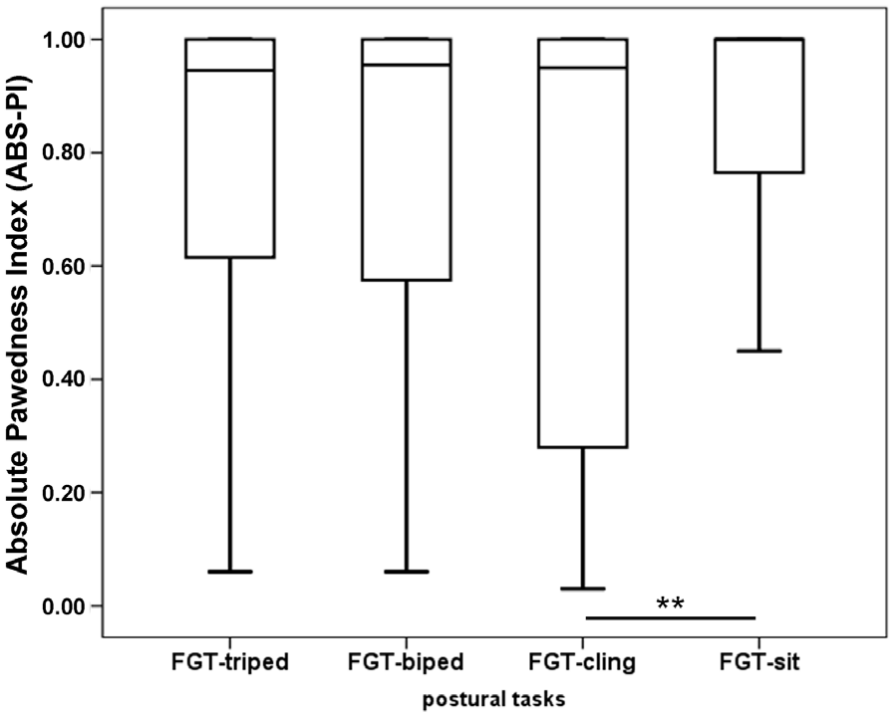
Mean Absolute Pawedness Index (PI) for the four postural tasks. The same individuals were tested in all four tasks (N = 28). ** Wilcoxon Test, p<0.01.

We compared the PI between the four postural tasks for the 28 subjects participating in all four tasks, but found no significant differences (Friedman test: Chi square = 2.7, df = 3, N = 28, p = 0.437, Figure 1). However, the ABS-PI tended to differ between the four postural tasks, (Friedman test: Chi square = 7.5, df = 3, N = 28, p = 0.055). Pairwise comparisons revealed a significant difference in ABS-PI between FGT-cling and FGT-sit (Wilcoxon-test: T = 7, n = 28, p = 0.005; Figure 2). The ABS-PI value was significantly greater in the FGT-sit than in the FGT-cling (mean ABS-PI_sit_ = 0.80±0.31; mean ABS-PI_cling_ = 0.69±0.39), this being due to the presence of 3 outliers. The number of lateralised versus non-lateralised subjects did not differ significantly between the four postural tasks (Cochran's Q = 5.6, df = 3, N = 27, p = 0.188) suggesting that posture did not influence the direction and strength of paw preference. Comparing the direction of paw preference 17 of 28 subjects showed a consistent paw preference for all four postural tasks (10 left-pawed; 7 right-pawed; Table 1). Only one subject switched the direction of paw preference from one task to another one. Four subjects showed a consistent paw preference for at least two tasks and were ambidextrous for the remaining tasks (Table 1). The PI and ABS-PI of the four postural tasks showed a significant positive correlation with one another (Spearman correlation for PI: rs≥0.838, N = 28, p<0.001 and ABS-PI: Spearman correlation: rs≥0.527, N = 28, p<0.004).

### Level of difficulty of the postural tasks

**Figure 3:**
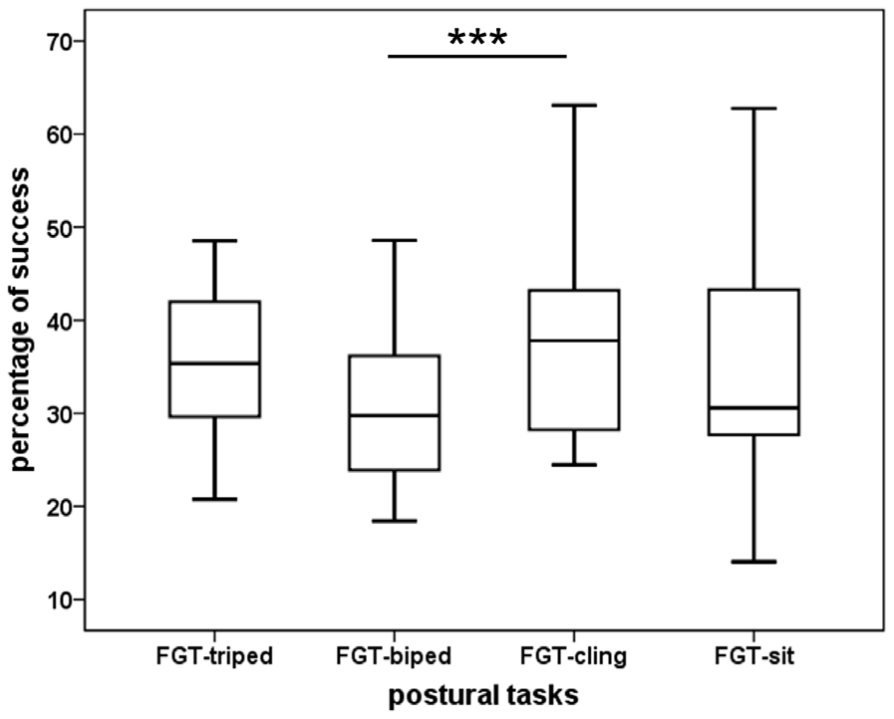
Mean success rate for the four postural tasks. The same individuals were tested in all four tasks (N = 28). *** Wilcoxon-test, p<0.001.

We calculated the percentage of successful grasps ( = success rate) to measure the level of difficulty of the postural tasks. The success rate differed significantly between tasks (Friedman-test: Chi Square = 17.24, df = 3, N = 28, p<0.001, Figure 3). Pairwise comparisons showed that the FGT-biped was significantly more difficult for the subjects than the FGT-cling (Wilcoxon-test: T = 9.25, n = 28, p<0.001; mean success rate_biped_ = 32.63±11.90%; mean success rate_cling_ = 39.23±12.39%).

## Discussion

We found that in the simple food grasping task (SGT), *i.e.* in a natural-like foraging situation, tree shrews prefer to use the mouth alone to pick up mealworms over using a paw-mouth combination or the paw alone. Nevertheless, when the use of the mouth was prevented, tree shrews showed the ability to grasp mealworms with one paw and showed an individual paw preference. No population level paw preference in all four postural tasks was found. We found no significant differences in the direction and strength of paw preference between the four postural tasks. The majority of subjects showed consistent paw preference in all postural tasks. Further, we found significant positive correlations for the direction of paw preference between the postural tasks. Although paw preference did not differ between the postural tasks, we found differences in their level of task difficulty, suggesting that grasping bipedally was more difficult for *T. belangeri* than grasping while clinging.

### Paw usage and grasping abilities

In the simple food grasping task, reflecting the natural foraging environment, tree shrews prefer to use the mouth than paws. It has been previously shown that mouth-foot, mouth-paw or, mouth-hand preferences may be actually linked to differences in foot/paw/hand function in foraging behaviour in tetrapods [12], [24], [25], [41], [42], [43], [44], [45]. Although behavioural observations of wild tree shrews are sparse, previous studies showed that according to the species, they may root and probe under the litter to find insects and also gain food by licking on exudates produced by the lid aerial pitcher of *Nepenthes* plants, processes not requiring paw usage [46], [47]. Among tree shrews, species which probe under the litter to find insects and thereby do not necessitate the use of paws, prefer to use the mouth over paws, while species foraging on fruits use the paws more than the mouth [48].

In the exact same simple food-grasping task, gray mouse lemurs used the hands in combination with the mouth more frequently [14]. Although mouse lemurs feed on gum [49], [50], [51], [52] by typically using their teeth to scratch tree bark and lick the gum and thus do not necessary require the hand to get food [51], they catch flying insects, which requires both hands, this maybe explaining the difference in mouth-hand usage compared with tree shrews. Mouth-hand usage can indeed be linked to different feeding strategies in nonhuman primates. As with mouse lemurs, dwarf lemurs [25], [42], greater galagos [25], [42], marmosets [24] and sifakas [41] used their mouth more than other nonhuman primates. Differences in mouth-hand preferences were linked to species differences in hand function in foraging behaviour even within the same family: marmosets predominantly pick up food items with the mouth, while lion tamarins, which are specialised in using manipulation and extracting insects, preferred the hand [24]. Similar results were found in non primate mammals. For instance, rats and opossums, both omnivores, preferred mouth over paws to pick up inanimate food items but can use only one hand to catch moving prey [43]. Within carnivores, species show a great variation in their forelimb usage and dexterity and it is correlated with phylogeny and estimated biomass of vertebrates in the diet [45]. Within non primate mammals, species-specific selective pressures are also found in the same genus. Frugivorous tree kangaroo species usually picked up food with their mouth while folivorous tree kangaroo species used their paws more [44]. The link between paw/hand function in foraging behaviour and mouth-paw or mouth-hand preferences therefore seems to be a universal pattern in tetrapods.

### Individual paw preference but no population bias in forced-food grasping tasks

We characterised for the first time the paw preference of a Scandentian species and demonstrated that most of the subjects show a paw preference for grasping food. The biased paw preference was however only found at an individual level and not at a population level. Our results corroborate with food grasping paw/hand preference at an individual but not at a population level in small-bodied primate species using the same test battery [14]. These findings tally with those of Rogers and Workman [53] who suggested that active use of paws or hands for feeding or searching for food is required for population level manual lateralisation. Both tree shrews (this study) and mouse lemurs [8], [14] demonstrate a poor paw usage or a limited hand usage while foraging which would, according to Rogers and Workman [53], also predict the lack of paw lateralisation at a population level.

### Task difficulty, postural instability and influence on manual laterality

In this study, using similar test batteries as in small-bodied primates, we revealed that postural demand did not influence the direction of paw preference in a small-bodied omnivorous mammal. Most of our subjects maintained their paw preference across the four forced-food grasping tasks, despite the difference in postural demand.

We did not find any significant difference in the strength of hand preference between the four forced-food grasping tasks. There was only a difference with a higher strength of paw preference in the FGT-sit than in the FGT-cling task which disappeared with the result of 3 outliers. These results do not support the bipedalism theory which proposed a significant increase in the strength of hand preference from a stable to an unstable posture [27]. A speculative explanation could be that it was more difficult for the subject to find an appropriate position for grasping a mealworm in the FGT-cling task, this thus influencing the strength of the paw preference. Even if the subjects had a preference for a paw (already determined in the FGT-sit and FGT-biped which were performed first), they sometimes tried to grasp with their non-preferred paw and the ABS-PI measure was therefore lower. This was not the case for the FGT-sit, since the animals were placed right in front of the box and the position of the body was restricted by the width of the wooden bar they were sitting on.

As introduced in another study [14], we used measures for estimating task difficulty to determine whether tasks of different postural demand varied in their level of difficulty for *Tupaia belangeri*. First, we found that in all four tasks subjects needed to grasp several times (a mean between 2 and 3 times corresponding to 30–40% success rate) to catch a mealworm. We thereby conclude that the FGT were quite challenging for tree shrews. Moreover, we found that the FGT-biped was more difficult to solve for *T. belangeri* than the FGT-cling task: subjects needed to grasp more to be successful in retrieving a mealworm. All subjects were tested successively in the FGT-triped, FGT-biped, FGT-cling and FGT-sit meaning that even if the postural demands differ, we can not rule out that subjects may have acquired more experience and therefore were more skilled in performing the FGT-cling than the FGT-biped which would result in a better success rate in the FGT-cling. However, both *Tupaia belangeri*'s morphology and posture stability may also explain this result. In the FGT-biped, tree shrews had to grasp while extending their hindlimbs. Although in the FGT-biped task they usually use one paw along the grid in front of the box to stabilise their body posture, we infer that it was more unnatural for them to stand than to grasp a mealworm while maintaining their position on the grid with one paw and two feet as in the FGT-cling task. As reported by Sargis [38], the forelimb of *Tupaia* is extended and adapted for terrestrial or scansorial locomotion. It is also less mobile in its joints, which restricts movement more to the parasagittal plane, thus optimising quadrupedal movements on the ground or on a regular substrate. The link between morphological adaptation and the level of difficulty measured in each postural task is supported by previous results in small-bodied primates performing the same postural tasks, using the same experimental procedure [14]. Indeed, contrary to tree shrews the comparable postural task FGT-sit was more difficult than the FGT-cling and FGT-biped postural tasks in those arboreal small-bodied primates [14]. In gray mouse lemurs, it was also argued that the different measured success rates could have also been a result of the different body movement axis used by a subject to grasp a mealworm or have been explained by the experience acquired in the previous forced-food grasping tasks [14].

To conclude, this study showed that in a natural foraging situation, a small-bodied rooting mammal prefers to use its mouth than its paws. Nevertheless, in a foraging task where mouth usage was prevented individual paw preferences were demonstrated, but no population-level paw preference independent of task-specific body posture. Our results support the hypothesis that a quadrupedal non primate mammal with a horizontal orientation to the trunk prefers mouth retrieval of food and shows no bias at a population level to use one hand and no influence of the postural demand on the strength of manual laterality. Yet, results reveal that postural demand has an influence on hand preference in some nonhuman primate species (e.g., [17], [20], [27]) but not in prosimians and tree shrews which share many features with primate ancestors (e.g., [14], [31]). Although future comparable studies using similar experimental procedures on other non-primate mammalian groups are crucial to explore to what extent our findings can be generalised, we suggest that influence of postural demand on paw/hand preference may not be linked to phylogenetic constraints but rather to ecological adaptation and possibly having evolved in large-bodied quadrupedal mammals specialised in fine manipulations of food item.

## Materials and Methods

### Ethics statement

The experiments were licensed by the Bezirksregierung Hannover, Germany (reference number: 509c-42502-03/ 660) and complied with the Animal Care guidelines and the applicable national law.

### Subjects

We tested a total of 36 northern tree shrews (*Tupaia belangeri*, 18 males, 18 females) of our breeding colony, housed in the animal facility of the Institute of Zoology, University of Veterinary Medicine Hannover (for details on housing conditions see [54], [55]. All subjects had been born in captivity. Their ages ranged from 1 to 9 years.

### Experimental set-up

**Figure 4:**
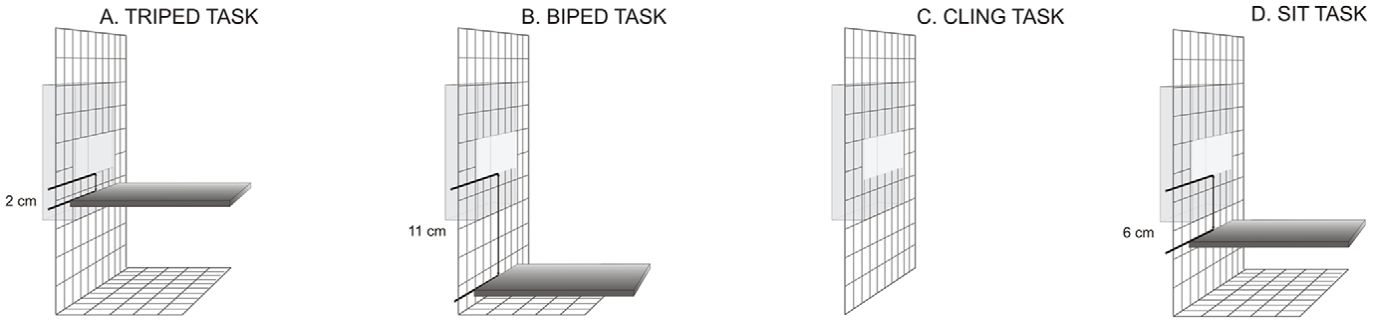
Experimental set-up for the four postural tasks (FGT-triped, FGT-biped, FGT-cling and FGT-sit). A plastic shield was used to standardise the position of the subject in front of the transparent box.

The experimental procedure was similar to [14]. Each tree shrew was tested alone in a test cage (Ebecco stainless steel cage, 50 cm×150 cm×80 cm) in a separate testing room. The cage was equipped with three wooden bars and a nest box. For the simple food grasping task (SGT), a food bowl (diameter: 10 cm) was placed in the test cage. For the forced-food grasping tasks (FGT), a transparent box with a small opening (2×4.5 cm) was attached to the outside of the cage (FGT-triped, FGT-biped, FGT-cling, FGT-sit; Figure 4). This prevented the animals from using their mouth so that they were forced to grab with one paw through the small openings between the bars. The subjects' behaviour was videotaped using a digital camcorder (SONY Camcorder DCR-SR55 HDD). The camera was connected to a monitor outside the testing room where the experimenter sat observing the subjects.

### General Procedure

Each session was conducted at the beginning of the activity period for each subject. For each session a subject was removed from its home cage, placed in a new nest box attached to the test cage in the testing room. For each session 10 mobile (SGT) or immobile mealworms (FGT) were placed in the food bowl (SGT) or plastic box (FGT). Each subject was tested for 20 minutes or until the subject had eaten all food items. A session started as soon as the door to the testing room had been closed to rule out any influence of the experimenter. An experimental task consisted of three sessions on three separate days. Thus, a subject needed a minimum of three days ( = three sessions) to complete one experimental task.

### Experimental tasks

#### Simple food grasping task (SGT)

In the SGT task, we collected data for familiar actions belonging to the natural repertoire of the subjects. For each session we scattered 10 living mealworms on the bottom of a food bowl and the subjects were allowed to pick up the food items either with their paws or with their mouth or with a combination of both (see Video S1). This task was performed by 14 tree shrews (7 males, 7 females).

#### Forced-food grasping tasks with variation in postural demands (FGT)

To test for the effect of postural demands we conducted four forced-food grasping tasks: FGT-triped, FGT-biped, FGT-cling, FGT-sit. In the FGT a subject had to use one of its paws to grab immobile mealworms (mealworms had to be immobilised to prevent them from crawling out of the transparent box) through a small opening (2×4.5 cm) in a transparent box (FGT-triped, FGT-biped, FGT-cling, FGT-sit; Figure 4). To induce different postural demands the transparent box was fixed at different heights to the wooden bar (Figure 4).

For the FGT-triped task, the opening of the transparent box was fixed at a distance of 2 cm from the wooden bar (see Figure 4 and Video S2). Thus, when the subject picked up a food item, both feet and one paw touched the ground while the other paw grasped the mealworm. This task was performed by 36 tree shrews (18 males, 18 females).

For the FGT-biped task the opening of the transparent box was fixed at a distance of 11 cm from the wooden bar (see Figure 4 and Video S3). The subject had to stand on its hind legs and stretch its body while manipulating the food items with both paws. This task was performed by 36 tree shrews (18 males, 18 females).

For the FGT-cling task the opening of the transparent box was fixed onto the grid of the cage (see Figure 4 and Video S4). The transparent box was positioned in such a way to prevent the subject from coming into contact with the ground while taking the food items. The subject had to cling onto the grid while manipulating the food items. This task was performed by 35 tree shrews (18 males, 17 females).

For the FGT-sit task the opening of the transparent box was fixed at a distance of 6 cm from the wooden bar (see Figure 4 and Video S5). The subject could sit on its hind legs while both paws were free. This task was performed by 29 tree shrews (13 males, 16 females).

For task comparison, data of 28 tree shrews (12 males, 16 females) which performed all four postural tasks were used.

### Data and video analysis

For analytical purposes, the recorded digital files were transferred to an external hard disk. We conducted a frame-by-frame analysis (25 frames/second) in The Observer XT v.9. (Noldus Information Technology, Wageningen, the Netherlands).

For the SGT task, we recorded whether the subject used its mouth alone, its paw alone or a combination of both. Mouth alone was defined as occurring when the subject picked up the mealworm without using its paws. The paws were either on the edge of the bowl or on the bottom with no contact to the food item. Paw alone was defined as occurring when the subject picked up the mealworm without using its mouth. That means the subjects transferred the food item to the mouth after the item was no longer in contact with the ground. A combination of paw and mouth was coded, if the two other behaviours were excluded, meaning subjects made a whole body movement and lunged at the food item with mouth and paws simultaneously. For the FGT tasks, we recorded the paw (right or left) the subject used to retrieve mealworms from the transparent box.

To measure the paw spontaneously chosen for a specific task ( = paw preference), we analysed the first grasp of each grasping bout. A grasping bout started with the first grasp of the subject and ended when it successfully retrieved a mealworm. A paw was considered to be successful when it had picked up one or more mealworms out of the box. A maximum of 20 grasping bouts ( = 20 mealworms) could be analysed per session. If the tree shrew retrieved one or more mealworms out of the box successfully, it ate them before starting a new grasp. Therefore, the first grasps of each grasping bouts can be considered as independent from each other.

Measurements on paw performance (analysis on paw which successfully retrieved a mealworm) were also performed but since the results did not differ from paw preference we presented the results for paw preference only.

### Statistical analysis

We calculated the pawedness index (PI) for each subject according to the formula PI = (number right−number left)/(number right+number left) [9]. The PI value can range from −1 to 1, with positive values reflecting right-paw bias and negative values reflecting left-paw bias. We additionally used the absolute PI (ABS-PI) value of each subject to compare the strength of the lateralisation irrespective of direction.

We tested whether subjects used one paw more often than expected by chance using the Binominal test with 50% chance level. We defined animals as left- or right-pawed or ambidextrous: right-pawed subjects used the right paw significantly more often than expected by chance (positive pawedness index), left-pawed subjects used the left paw significantly more often than expected by chance (negative pawedness index), ambidextrous subjects did not use one paw significantly more often than expected by chance. We also calculated the Z-score and found the same results as using the Binomial test. In the result section, we therefore presented only the results of the Binomial test.

According to a Kolmogorov-Smirnov test, our data differed significantly from a normal distribution. For this reason, we used nonparametric tests (two-tailed). To explore whether a significant majority of the population was lateralised, we used a Chi-Square test with the number of left-, right-pawed, and ambidextrous individuals to test if this distribution differed significantly from chance (25∶25∶50, [1]). To test if the population showed a lateralisation towards the right or the left paw, a binomial test was conducted to test whether significantly more subjects used the right paw than expected by chance (50∶50). Additionally, we performed a one-sample t-test on the PI score to investigate pawedness at population level as is commonly done in the literature [56].

To explore sex differences we compared the PI and ABS-PI of males and females, using the Mann-Whitney- U test. We explored age effects by correlating the PI and ABS-PI with the age of the subjects, using a Spearman correlation.

To investigate the effect of postural demands we compared the PI and ABS-PI between the four postural tasks, using the Friedman test. Further, we compared the number of lateralised subjects between the four postural tasks, using the Cochran's Q test. We used the Spearman correlation to examine the relationship between the PI and ABS-PI for the four postural tasks. To evaluate the level of difficulty of the postural demand tasks we calculated the percentage of successful paw grasps by dividing the number of successful paw grasps by the total number of paw grasps ( = success rate). A success rate of 100% meant that the subject was successful in all grasps. A success rate of 50% meant that the subject successfully retrieved a mealworm in only half of all grasps. We compared the level of difficulty between the four postural tasks using the Friedman test. All statistical tests were exact and calculated using PASW Statistics 18 (previously SPSS; IBM Company). We considered a result significant if p≤0.05.

## Supporting Information

Video S1 Example of an experimental trial of the simple food grasping task (SGT).
(MPG)

Video S2 Example of an experimental trial of the FGT-triped task.
(MPG)

Video S3 Example of an experimental trial of the FGT-biped task.
(MPG)

Video S4 Example of an experimental trial of the FGT-cling task.
(MPG)

Video S5 Example of an experimental trial of the FGT-sit task.
(MPG)
